# Dysphagia caused by a fibrovascular polyp: a case report

**DOI:** 10.1186/1757-1626-1-334

**Published:** 2008-11-19

**Authors:** Marielle MJ Blacha, Cornelius EJ Sloots, Ivo P Van Munster, Theo Wobbes

**Affiliations:** 1 Department of Surgery, Radboud University Nijmegen Medical Center, the Netherlands; 2 Department of Gastroenterology, Jeroen Bosch Hospital, 's-Hertogenbosch, the Netherlands

## Abstract

A 73-year old man presented with dysphagia for liquid and solid food. Barium contrast study of the esophagus and esophagoscopy demonstrated a fibrovascular polyp. This, almost 10 cm benign esophageal tumor, was removed surgically by a cervical esophagotomy. A fibrovascular polyp is a rare benign tumor of the esophagus, which, however, may give serious complications as asphyxia resulting from laryngeal obstruction leading to sudden death.

## Introduction

Dysphagia is a serious symptom and it can be caused by malignant process such as esophageal cancer or compression of the esophagus, neurological and motility disorders such as achalasia, or benign disorders like strictures, diverticula or esophagitis. However, seldom dysphagia is caused by a benign polyp, which protrudes in the esophagus.

## Case presentation

**Figure 1:**
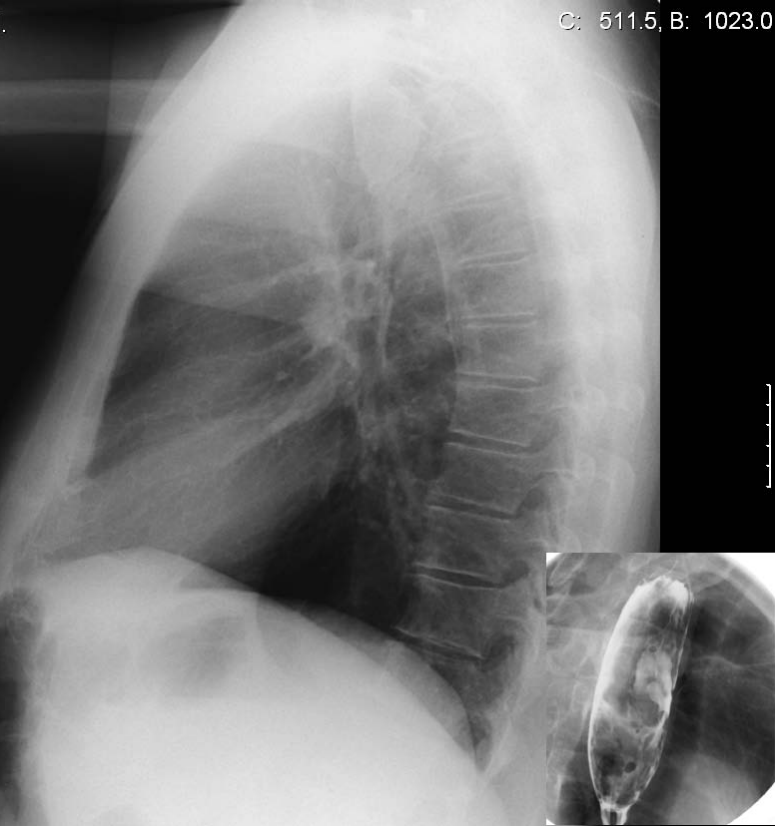
**Lateral chest x-ray showing a shadow ventrally of the vertebra in the posterior mediastinum.** Insertion shows a barium swallow with a large tubular intraluminal mass in the proximal esophagus.

**Figure 2:**
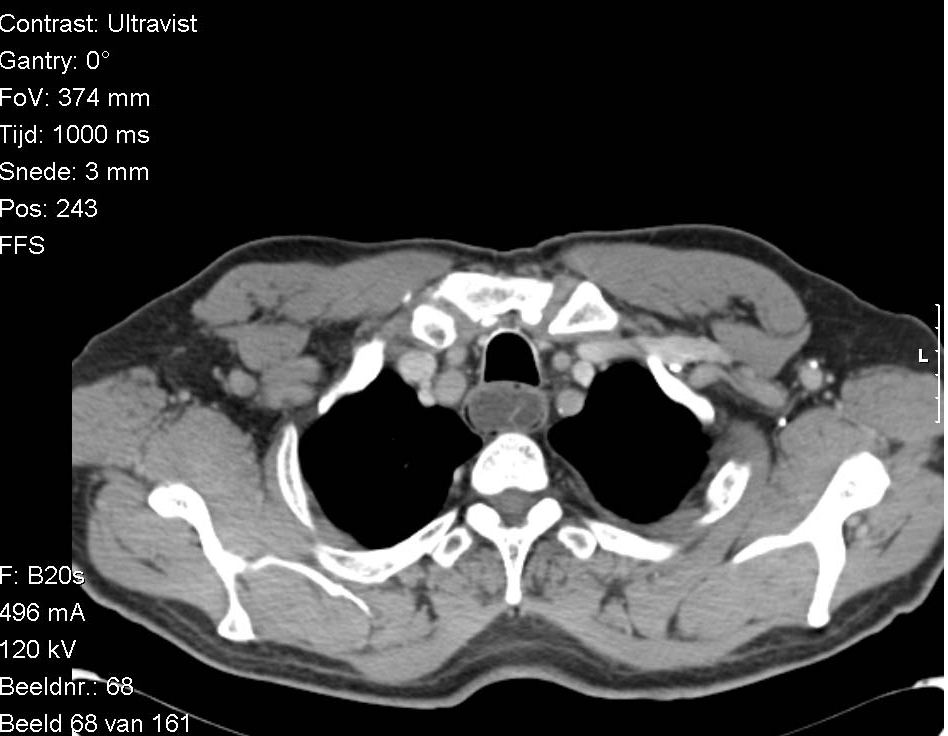
CT scan showing the large polyp in the esophagus nearly occluding the lumen.

**Figure 3:**
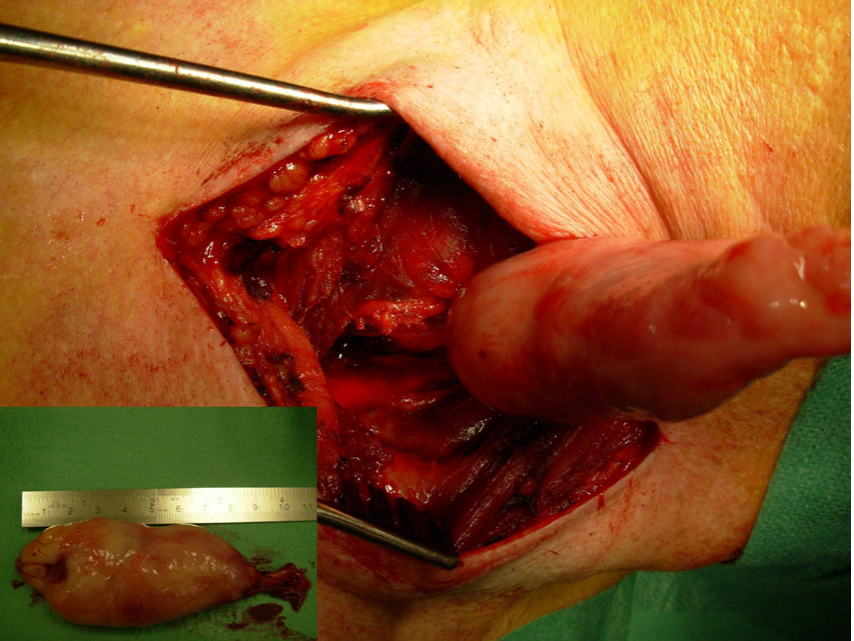
Pulling out the pedunculated polyp via a transcervical esophagotomy. Insertion shows the almost 10 cm long polyp.

A 73-year old male patient presented with dysphagia that existed for about four months. He reported the sensation of a mass in his throat during swallowing of solid as well as liquid food. The complaints suggested an intraluminal tumor. Barium contrast study demonstrated a tubular intraluminal mass originating from the proximal esophagus distal to the crico-esophageal junction (Figure 1). Esophagoscopy showed a smooth mass in half of the esophageal circumference. The polyp was attached to the hypopharynx on the right. On the lateral chest x-ray, the mass was already visible in the posterior mediastinum (Figure 1). Computed tomography scanning of the chest showed an intraluminal pedunculated lesion in the upper to middle esophagus (Figure 2). The radiographic and esophagoscopic findings were suggestive for a fibrovascular polyp. In order to remove the polyp, the patient underwent a left-sided cervical esophagotomy. The mass was pulled out via a length esophagotomy. The pedicle was ligated at the right side of the esophagus and the polyp extirpated (Figure 3). Postoperatively, the patient complained of dysphagia for solid food and dysphonia, probably due to edema at the esophagotomy wound. Six weeks postoperatively, all symptoms had disappeared.

Macroscopically, an almost 10 cm sausage-shaped mass was removed (Figure 3). Histological examination revealed a polypoid lesion covered by squamous epithelium. The underlying stroma consisted of a mixture of large vascular structures, fibrous connective and myxoid tissue. No signs of inflammation, dysplasia or malignancy were identified. These features match with the diagnosis of a fibrovascular polyp.

## Discussion

Benign tumors of the esophagus account for 20% of the esophageal lesions. 60–80% of the lesions are leiomyomas, typically occurring as intramural lesions in the middle and lower esophagus [1]. The second most common benign esophageal tumors are squamous papillomas. They occur as small finger-like polyps in the lower esophagus. Hemangiomas of the esophagus are rare, but more prevalent than fibrovascular polyps [2]. Fibrovascular polyps, or allegedly polypoid fibroma, myoma, fibrolipoma, pedunculated lipoma, fibroepithelial polyp or lipoma of the hypopharynx, account for 1–2% of all esophageal tumors [5]. They are most commonly found in males around the age of 50 years [2].

Fibrovascular polyps are postulated to originate from two areas of lower resistance in the pharyngeal musculature. One is between the superior and inferior cricopharyngeal muscle (Killian's dehiscence), the other is between the inferior cricopharyngeus muscle and the proximal end of the esophagus (the Laimer's triangle). In these areas, nodular submucosal thickenings of submucosal folds may evaginate through the mucosa. Due to peristaltic activity and traction the evagination increases to a giant polyp up to 25 cm in length [3]. Histopathologically, the polyp exists of fibrovascular connective tissue with fatty cells and is covered with squamous epithelium [4].

Since the polyp is slowly growing, it may become symptomatic after years. The most common complaints include dysphagia and sensation of a mass. Other complaints are retrosternal or epigastric discomfort, odynophagia, vomiting, weight loss and respiratory symptoms such as persisting cough and shortness of breath. The most distinctive feature of a fibrovascular polyp is regurgitation into the mouth [2]. A feared complication is asphyxia and laryngeal obstruction by the polyp causing sudden death [5,6]. When the polyp twists, it leads to hemorrhage and necrosis of the lesion. Hematemesis or occult gastrointestinal bleeding leading to anemia is probably related to ulceration of the tip of the polyp due to peptic digestion when it protrudes through the cardia into the stomach. Although unusual, the polyp can show malignant degeneration [2].

Fibrovascular polyps are visualized by different modalities such as barium contrast studies, esophagoscopy, endosonography, CT scanning and MRI. A barium contrast study typically demonstrates a significant intraluminal-filling defect within the widened esophagus, from the upper esophagus till the gastro-esophageal junction, while on swallowing the defect moves along. The polyp is shown by endoscopy, however, since it is covered with normal mucosa, it is easily missed. Chest x-ray shows a posterior mediastinal mass. CT scanning, MRI or ultrasonography can be useful to diagnose the fibrovascular polyp. In particular, MRI might be decisive in the choice of treatment by demonstrating the composition of the polyp. If the mass consists predominantly of fat with a minimal blood supply, the risk of bleeding during an endoscopic treatment is small. In case the polyp is rich of vascular structures, endoscopic resection can be troublesome due to uncontrollable bleeding [7]. Therefore, the first choice of therapy is surgical excision, preferable by a left-sided cervical approach. In case of a large sized polyp a thoracotomy may be necessary. Possible complications of a cervical polypectomy are (transient) dysphonia, pneumothorax and development of a cervical esophagus fistula [8]. Recurrence of the lesion is only seen when residual tissue is left at the resection site [2].

## Conclusion

A fibrovascular polyp is a seldom-occurring benign tumor of the esophagus causing dysphagia. It is to be removed by surgical excision preventing serious complications such as asphyxia and laryngeal obstruction.

## Consent

Written informed consent was obtained from the patient for publication of this case report and accompanying images. A copy of the written consent is available for review by the Editor-in-Chief of this journal

## Competing interests

The authors declare that they have no competing interests.

## Authors' contributions

MMJB was first author. CEJS was involved in collecting pictures, performing surgeon and was the second author. IPVM was involved in case preparation and was the referring gastroenterologist. TW was the performing surgeon and was involved in supervision
